# Innovations to the ECHO model to enhance reach and network-building among addiction clinicians in Western Canada

**DOI:** 10.1186/s13722-024-00524-z

**Published:** 2024-12-18

**Authors:** Samantha Robinson, Isabella Brohman, Jenna van Draanen, Rivka Kushner, Nadia Fairbairn, Stephanie Glegg

**Affiliations:** 1 British Columbia Centre on Substance Use, 400 – 1045 Howe Street, Vancouver, BC V6Z 2A9 Canada; 2 School of Nursing, University of British Columbia, Westbrook Mall, Vancouver, BC T201 –2211, V6T 2B5 Canada; 3 School of Public Health, Department of Health Services, University of Washington, 3980 15th Ave NE, Fourth Floor, Box 351621, Seattle, Washington, Seattle, WA 98195 USA; 4 School of Nursing, Department of Child, Family, and Population Health Nursing, University of Washington, 1959 NE Pacific Street, Box 357263, Seattle, Washington, Seattle, WA 98195-7263 USA; 5 Faculty of Medicine, Department of Medicine, University of British Columbia, 2206 East Mall, Vancouver, BC V6T 1Z3 Canada; 6 Faculty of Medicine, Department of Occupational Science and Occupational Therapy, University of British Columbia, T325 – 2211, Westbrook Mall, Vancouver, BC V6T 2B5 Canada

**Keywords:** Addiction, Education, ECHO, Innovations, Adaptations, Opioid use disorder, Alcohol use disorder, Substance use

## Abstract

**Background:**

Building capacity for evidence-based treatment and support for people with substance use disorders (SUD) is an urgent priority in the context of the toxic drug poisoning crisis. We implemented the first substance use-focused Project Extension for Community Healthcare Outcomes (ECHO) in Western Canada for health care providers, to enhance their clinical addiction skills and knowledge, facilitate practice change, and foster a supportive community of practice. The aims of this article are to describe our innovations to the Project ECHO model in British Columbia (BC) and Yukon, and present key program outcomes.

**Methods:**

A pragmatic multi-methods program evaluation employed observational records of BC ECHO on Substance Use session attendance, cross-sectional and longitudinal participant surveys, and qualitative interviews with participants to assess satisfaction, relevance, and preparation to use evidence-based approaches, practice change intentions, and actual behaviours.

**Results:**

The 52 ECHO sessions (from June 2019 to July 2022) attracted 2134 unique registrants with 5089 attendances (mean 124/session), 2132 newsletter subscribers, and 5842 podcast downloads. The evaluation included 844 post-session survey respondents and 53 interview participants. The program included ECHO sessions with rolling attendance; widely accessed supplemental formats (e.g., newsletter, podcast, clinical tools, archived presentation recordings); variable, regional hub representation; and evidence-based content developed by medical writers. These features contributed to broad geographic and discipline reach, high-quality program content, and high mean session satisfaction ratings (4.2/5). Key qualitative themes emerged, related to knowledge and skill acquisition, gaining confidence in providing SUD care, facilitating shared decision-making, increasing compassion for patients, consolidating learning and applying it to practice, and reducing isolation through expanded networks.

**Conclusions:**

The ECHO model is an effective way to improve capacity in SUD care for physicians and nurse practitioners, while offering benefits for interprofessional attendees. Our findings can inform innovations in other ECHO programs to enhance reach, engagement, and impact.

**Supplementary Information:**

The online version contains supplementary material available at 10.1186/s13722-024-00524-z.

## Background

The recent increase in opioid-related mortality, driven largely by the emergence of potent, toxic, synthetic opioids (i.e., fentanyl and fentanyl analogues), has had devastating impacts, even slowing life expectancy growth in Canada and the US [1, 2]. Alcohol use is also a serious problem in North America, contributing to almost 200 disease states or injuries [3]. Globally, alcohol-related burden of disease is estimated to be two to three times higher than that of all illicit substances combined [3, 4].

Despite the burden of substance use disorders (SUD) on health systems, few primary care providers have incorporated evidence-based SUD care into their practices [4], leaving most people who would benefit from treatment without access [5, 6]. Recent steps to address barriers include developing clinical guidance, practice tools and online education for opioid use disorder (OUD) and alcohol use disorder (AUD), the removal of the requirement for federal exemption for methadone prescribing, and expanded treatment options (e.g., additional formulations of buprenorphine, injectable opioid agonist treatment) [5]. Despite these advances, the lack of standardized and accessible training among physicians and other providers to adequately screen, assess and treat patients with SUD hinders quality care [7].

Project Extension for Community Healthcare Outcomes (ECHO) is a low-barrier, high-impact, case-based continuing education model that aims to build competence and confidence among health care providers to deliver evidence-based care for a given area of medicine that has traditionally been specialist-led [8] Project ECHO uses a virtual “hub” and “spoke” model to connect an interdisciplinary group of clinicians with expertise in the area of focus (the “hub”) with several providers or teams (the “spokes”). The ECHO model has had broad reach globally and has contributed to improved access to health care in rural settings [9]. It has been proven to be a valuable tool for engaging and educating health care providers, with a recent systematic review demonstrating satisfaction, increased knowledge and clinical confidence among participants across several programs and disciplines [10]. Emerging evidence suggests that the model may contribute to improved patient health outcomes, and to a lesser extent, community outcomes [11]. Several Project ECHO programs target mental health and substance use care specifically, and evaluations of these programs have demonstrated positive changes among participants, including improved knowledge and self-efficacy in treating OUD [12, 13]. In addition, the implementation of ECHO has contributed to increases in the number of physicians who met the requirements to prescribe buprenorphine [13] improving access. As the case for primary care management of SUDs builds, there are increasing calls to implement innovative education approaches, such as ECHO, to bridge the knowledge-to-practice gap in addiction care among interprofessional primary care providers [14].

The British Columbia (BC) Centre on Substance Use (BCCSU) is a provincially networked organization with a mandate to develop, help implement and evaluate evidence-based approaches for substance use and addiction. Its three core functions are research; education and training; and clinical care guidance. Under the latter two streams, in 2019, we developed and implemented the first substance use-focused Project ECHO in Western Canada to: (1) Reach prescribers and other health care providers in BC and Yukon (urban and rural settings); (2) Equip participants with the knowledge, skills, and supports to manage OUD and/or AUD effectively; (3) Foster evidence-informed OUD/AUD program, policy or practice change; and (4) Foster a community of practice for ongoing clinical support for SUD care. The purposes of this article are to: (a) Describe the implementation of an adapted Project ECHO model in BC-Yukon; (b) Highlight innovations employed; and (c) Present key program outcomes.

## Methods

### Project ECHO model overview

Project Extension for Community Healthcare Outcomes (ECHO) is a low-barrier, case-based continuing education model based out of the University of New Mexico [8] Project ECHO traditionally has used a “hub” and “spoke” model to connect an interdisciplinary group of clinicians with expertise in the area of focus (the “hub”) with several providers or teams (the “spokes”). The model consists of virtual sessions that bring the hub and spokes together for a short didactic presentation on a given topic, followed by an anonymized case presentation by one of the spokes, case discussion, and collaborative recommendation development. The standard model comprises initial training on the ECHO model for participants ahead of session participation, two-hour duration for sessions, weekly session frequency and the combination of didactic and case presentations at each session. In the standard model, spokes usually commit to participating in a closed series (a set number of ECHO sessions) and agree to attend all sessions of the series, bring forward at least one real patient case for discussion, and actively contribute to case discussions. Session content is often developed by the hub team. Sessions afford collaborative learning and capacity building through the development of a community of practice.

### Program overview and adaptations

Adaptations were made to the standard ECHO model in response to feedback and direction from the Steering Committee (who met at least annually) and program participant evaluation data. We took a continuous quality improvement approach to implement adaptations as feedback was received.

#### Format and content

The OUD stream launched in June 2019 and the AUD stream launched in August 2020. Session frequency was typically bi-weekly. The two streams operated independently and on different days; a common website hosted information about both. Participants were invited to join any session from both streams. Sessions were scheduled in a series or ‘cycle’ with the goal of each cycle to cover a range of topics – with each session covering a different aspect of care, as outlined in the current provincial clinical guidelines (i.e., screening, pharmacotherapy approaches, urine drug screening, transitions between pharmacotherapies, approaches for specific populations, etc.). Session topics within a given cycle were unique. Instead of a consistent curriculum (standard model), our session topics evolved over time in response to identified needs. For example, subsequent cycles contained some repeated topics (especially for foundational content) and some new content, based on survey-identified learning needs or updated clinical guidelines. Cases were either real clinical scenarios submitted and presented by participants or composite cases that the team assembled when no clinical case was submitted. The team received participant feedback that some of the real cases contained too many complexities (e.g., multiple medical comorbidities, social challenges such as housing, etc.) and so simplified cases were developed to reflect real but pared down or composite clinical scenarios. These cases could reinforce core concepts from the didactic presentation with moderate complexity. Case recommendations were distributed after each session, and we obtained continuing medical education accreditation for the didactic session content. As the program evolved, we moved from only holding live sessions (standard model) to posting the didactic recordings online, to increase access. To further increase accessibility and knowledge uptake, sustain interest, and reach clinicians in diverse ways, we created several additional products, including monthly newsletters to share key program updates; blog posts highlighting participants and their practice and experiences with ECHO; clinical tools; and a podcast. The podcast, ‘Addiction Practice Pod’, provided clinical tips and several perspectives on topics that were too complex to cover in-depth during sessions. Many topics evolved from session discussions and case characteristics. The program website housed these resources, which were optional for participants; topics are available in Additional file 1.

#### Participants

Traditionally, the ECHO model reaches relatively small cohorts of health professionals who commit to be “spokes” (i.e., participate in sessions, contribute to case discussions, submit cases) for a set number of sessions. Given the ongoing provincial public health emergency related to the toxic drug poisoning crisis and the need to rapidly upskill interprofessional primary care providers in SUD care, registration remained open and rolling throughout the program, allowing session-by-session registration rather than commitment to every session, to maximize reach and remove participation barriers. Participants were recruited through targeted outreach by Steering Committee members to their regional teams and colleagues. Information about the program also appeared on the education section of the BCCSU’s website. The BCCSU was the primary source of provincial clinical education on substance use and substance use disorders, so participants may have learned about the ECHO program while visiting the website to access current clinical guidelines or for practice updates. Further, the ECHO was advertised through other education opportunities provided by the BCCSU, such as in-person seminars, and at the end of online education programs. Once registered on the website, participants could opt-in freely to any of the program components (i.e., ECHO sessions of any stream, newsletters, podcast, etc.).

#### Program staff

The clinical project coordinator or program manager for each stream oversaw day-to-day operations and planning. A project coordinator supported administration for both streams. A knowledge translation coordinator was responsible for the podcast and supported program communications (i.e., newsletter, blogs). The communications lead supported program promotion, web and podcast development. The clinical director provided overall leadership. The evaluation team conducted the evaluation. Medical writers developed the didactic presentations and clinical tools. Conversely, in the standard model, hub team members or the program medical lead are usually responsible for creating and delivering didactic content.

#### Advisory

A Steering Committee met at least annually to inform program development and curriculum planning and to support regional promotion. Members included clinical addiction leaders from the five regional Health Authorities, representation from the Yukon Department of Health and Social Services, and others from relevant health service organizations, including Doctors of BC. Committee consultation and consensus determined that a schedule with short, frequent lunch-hour ECHO sessions would best meet the needs of busy fee-for-service prescribers.

#### Hub team

Instead of one hub team comprising interdisciplinary clinicians, which is standard for the ECHO model, we engaged regional representation wherever possible. Clinical experts were diverse, varied by session, and often joined remotely. This approach differs from the standard model where a core group of experts join on a regular basis. One key challenge in developing and delivering a novel education program in the context of dual public health emergencies (the overdose crisis and the COVID-19 pandemic), was securing hub team members with substance use disorder expertise. Most leaders in addiction medicine held many different roles – academic, leadership, clinical, and had limited capacity to be involved. The session facilitator was usually a registered nurse with SUD treatment experience; a pharmacist with a large addiction practice also joined regularly, as well as at least two physicians with clinical addiction expertise to support recommendation generation and learning. As often as possible, a person with lived/living experience of SUD care joined the hub team to share their expertise as well. This interdisciplinary approach ensured relevance for participants from a range of primary care roles, such as physicians, nurses, nurse practitioners, pharmacists, social workers, team leads, and educators. Didactic presenters for a given cycle were selected from each region to encourage provincial uptake, spotlight local addiction medicine leaders, and enable discussions and case recommendations that would better reflect local contexts and available services. The hub team supported case discussions and reviewed the final sets of case recommendations. In this way, the program served a diverse Western Canadian audience and by having regional representation through hub team members and didactic presenters, urban/rural and regional differences and issues could be discussed.

### Study design

This pragmatic, multi-methods program evaluation employed observational records of BC ECHO on Substance Use session attendance, cross-sectional and longitudinal participant surveys, and qualitative interviews with participants. The research questions were:

1. What was the reach and satisfaction (with content, format, interactivity, session pace) of the ECHO program elements in terms of meeting participants’ needs?
2. What was the impact of the ECHO program elements on self-reported measures of knowledge, skills, confidence, and supports for participants?
3. How did the program contribute to participant-reported changes to practice, programs or policy?
4. To what extent did ECHO foster a community of practice?

By answering these questions, we aimed to highlight the benefits of the ECHO model and its specific adaptations to provide insight into others who may be considering adapting the ECHO model to meet the needs of their local context or fields, as well as to accommodate the constraints imposed by broader societal contexts, such as public health emergencies that present significant barriers to engagement in continuing professional development opportunities.

### Recruitment

ECHO participants were recruited through the BCCSU network, regional Health Authorities and the BC physicians’ association (Doctors of BC) via websites and emails/newsletters. Program and session registration occurred online. Session registration triggered an invitation to consent to program evaluation. All session registrants were emailed pre-session surveys via the registration process, and post-session surveys via email or in-session polling.

A random number generator was used to invite participants from the BC ECHO on Substance Use attendee list who had attended at least one OUD or AUD ECHO session to complete an interview. Interview invitations were emailed until saturation was achieved, determined by the research team’s consensus when no themes were presenting from consecutive interviews. Prescribers, participants who attended a recent ‘cycle’ of OUD or AUD ECHO, and/or participants who had attended 5 or more ECHO sessions were prioritized for initial invitations, although participants with a range of engagement were targeted to understand a range of perspectives to inform program improvements. Participants included nurses, nurse practitioners, pharmacists, physicians, social workers, clinicians, team leads, nurse educators, and health care consultants from all health regions in BC. Written consent preceded interviews; participants received a $50 honorarium.

### Data gathering

A custom-built online platform gathered demographic information at initial engagement with the ECHO program via an online form. Data included gender, organization, health authority, practice setting, city, professional role, region type [rural vs. urban], years of experience, and post-clinical entry education/training to describe the sample, and to enable participant engagement planning and reporting to funders on their key attributes of interest. Subsequent online session registration gathered name and email address to enable distribution of session meeting links to reduce respondent burden. Staff tracked attendance via spreadsheet. Participation in all other evaluation activities was optional to support low-barrier access to the program.

Survey data were gathered periodically throughout the program to coincide with the delivery of ECHO sessions and podcast episodes. Surveys are provided in Additional file 2. Pre-session surveys were developed by the team and sent to session registrants to understand what participants hoped to gain from the session, and their baseline ratings of confidence with the learning objectives. Post-session surveys sent to attendees immediately after the sessions repeated these rating items to evaluate change in confidence with the learning objectives, alongside items rating learner satisfaction with delivery, utility, relevance, format, and effectiveness at meeting learning objectives and expectations, to inform program improvements. Participants also rated their preparation to use evidence-based approaches, and intention to change practice as proxies for future practice change. All ratings used a 5-point Likert scale. Open-text responses probed planned practice changes and barriers to implementing them, to inform strategies to support practice change. Data collection took place between June 2019 and July 2022.

Podcast listeners, regardless of ECHO registration status, were invited to complete internally developed online podcast outcome evaluation surveys via web link on each episode’s landing page directly after listening, while content was fresh in their minds. Ten items tested knowledge of content (to assess retention) and asked about perceived benefits of listening, episode-driven intentions to change practice, and changes in SUD care knowledge and confidence, and open-text questions about the nature of anticipated practice changes, and feedback to inform podcast improvements. These short-term outcomes were deemed most feasible to assess based on the cross-sectional timing of the anonymous survey, for which follow-up was not practical.

Semi-structured, 45-60-minute individual interviews were completed with 28 OUD and 25 AUD stream participants from June-October 2020 and July-October 2021, respectively. This timing coincided with the delivery of cycle 3 sessions so that participants would have had time to engage adequately with the program. An interview guide for each stream was developed by the research team and the BC ECHO on Substance use team to address aspects of all four research questions (see Additional file 3). Questions probed program perceptions and impact on clinical practice in more depth, experiences in providing SUD care, and knowledge and training of AUD/OUD care to help the team tailor ECHO sessions to clinicians’ needs. Two research assistants who identified as women (IB, NA) and were trained in qualitative interviewing conducted the interviews using videoconferencing software. Interviews were audio recorded and professionally transcribed, and de-identified and checked for accuracy by a member of the research team.

### Data analysis

Quantitative data (demographics, participant ratings) were analyzed using descriptive statistics, including frequency counts, proportions, ranges, and means. Open-text responses were analyzed using conventional content analysis to derive categories based on text from the data [15]. Interview audio recordings were transcribed verbatim and imported into NVivo12 for analysis. An inductive and iterative thematic analysis was conducted. Two team members (IB and JvD) used a line-by-line method to inductively code transcripts and identify themes and sub-themes, and to develop and later refine a coding framework. Discrepancies in coding were resolved using a consensus-based approach. Once the interviews were completed, three team members (IB, JvD, SR for OUD/SG for AUD) with diverse backgrounds in public health policy, substance use research, and nursing/occupational therapy discussed the key themes and further refined the coding framework to include additional codes (e.g., impact of the COVID-19 pandemic on SUD care). Although participants did not engage in the interpretation or analysis of the data, the development of themes was guided by their input, ensuring that both shared experiences and diverse perspectives were captured.

## Results

The OUD stream launched in June 2019, with 39 sessions delivered by June 2022. The AUD stream launched in August 2020 and delivered 14 sessions (including one joint AUD/OUD session) by July 2022.

### Evaluation findings

Baseline survey respondent, session attendee and interview participant demographics are presented in Table 1. 50% of session registrants were nurses (including registered nurses, nurse practitioners and other nursing roles); physicians and social workers were the next most represented professions. Twenty-eight OUD and 25 AUD session participants were interviewed from June-October 2020 and July-October 2021, respectively and had attended a mean of 6 (SD 2.7, range 1 to 13) sessions. Registered nurses, nurse practitioners, physicians and social workers comprised the majority of interview participants. The majority of individuals in all samples identified as women, while a quarter of session participants were from rural regions. The podcast (*n* = 17) surveys were anonymous.

**Table 1:** Participant demographics

Demographic characteristics	# (%) of participants		
**Profession**	**Baseline Survey**	**Session Registration** ^**Ψ**^	**Interviews**
Physicians	121 (16%)	239 (11%)	6 (11%)
Nurse practitioners	65 (9%)	110 (5%)	10 (19%)
Registered nurses	217 (28%)	472 (22%)	13 (25%)
Other nursing roles	8 (1%)	502 (23%)	0 (0%)
Pharmacists	15 (2%)	104 (5%)	8 (15%)
Social workers	59 (8%)	238 (11%)	6 (11%)
Other clinicians*	92 (12%)	128 (6%)	3 (6%)
Support worker roles^Δ^	36 (5%)	82 (4%)	0 (0%)
Students, other and unknown	133 (18%)	280 (13%)	7 (13%)
**Gender**	**Baseline Survey**	**Session Registration** ^**Ψ**^	**Interviews**
Woman	481 (64%)	765 (36%)	51 (96%)
Man	96 (13%)	167 (8%)	2 (4%)
Two-spirit, transgender, non-binary	6 (1%)	12 (< 1%)	0 (0%)
Declined to answer	163 (22%)	1211 (56%)	0 (0%)
**Geographic region**	**Baseline Survey**	**Session Registration** ^**Ψ**^	**Interviews**
Urban	437 (59%)	975 (45%)	26 (49%)
Rural	245 (33%)	506 (24%)	17 (32%)
Other (includes ‘not applicable’, ‘neither’, ‘unsure’ and unknown)	64 (8%)	674 (31%)	10 (19%)
**Total n**	**746**	**2155**	**53**

Note: *Other clinicians=“clinician” (36), counsellor (23), occupational therapist [12], mental health/substance use clinician [12], paramedic [2], paraprofessional in social work [1], recreation therapist [1], “therapist” [1], “allied health professional” [1], concurrent disorders clinician [1], physiotherapist [1], dentist [1]; ^Δ^Support worker roles = outreach worker [10], community support/residential worker [7], wellness worker [6], mental health worker/care aide [5], recovery support worker [2], special services worker [2], social development support worker [1], youth addiction support worker [1], family peer support worker [1]. ^Ψ^Session Registration demographics are a proxy for post-session survey respondent demographics, as post-session surveys did not gather demographics, and approximately half of the data have been anonymized and cannot be linked to registrant demographics

### Objective 1: Access to and satisfaction with content

#### Reach

**Fig. 1:**
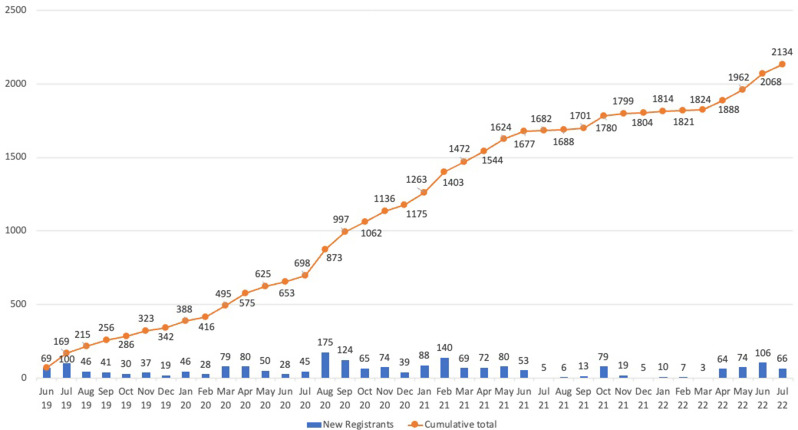
Monthly ECHO registrants since launch of the program

The 2155 unique OUD and AUD ECHO registrants from June 27, 2019 to July 31, 2022 combined for a total of 5089 attendances across the 52 unique sessions (mean 124 participants/session). Demographics are provided in Table 1. Program registration rates since launch are depicted in Fig. 1. As of July 2022, the newsletter distribution list had grown to 1147 individuals; the 12 podcast episodes had a combined 5842 downloads.

#### Satisfaction

**Fig. 2:**
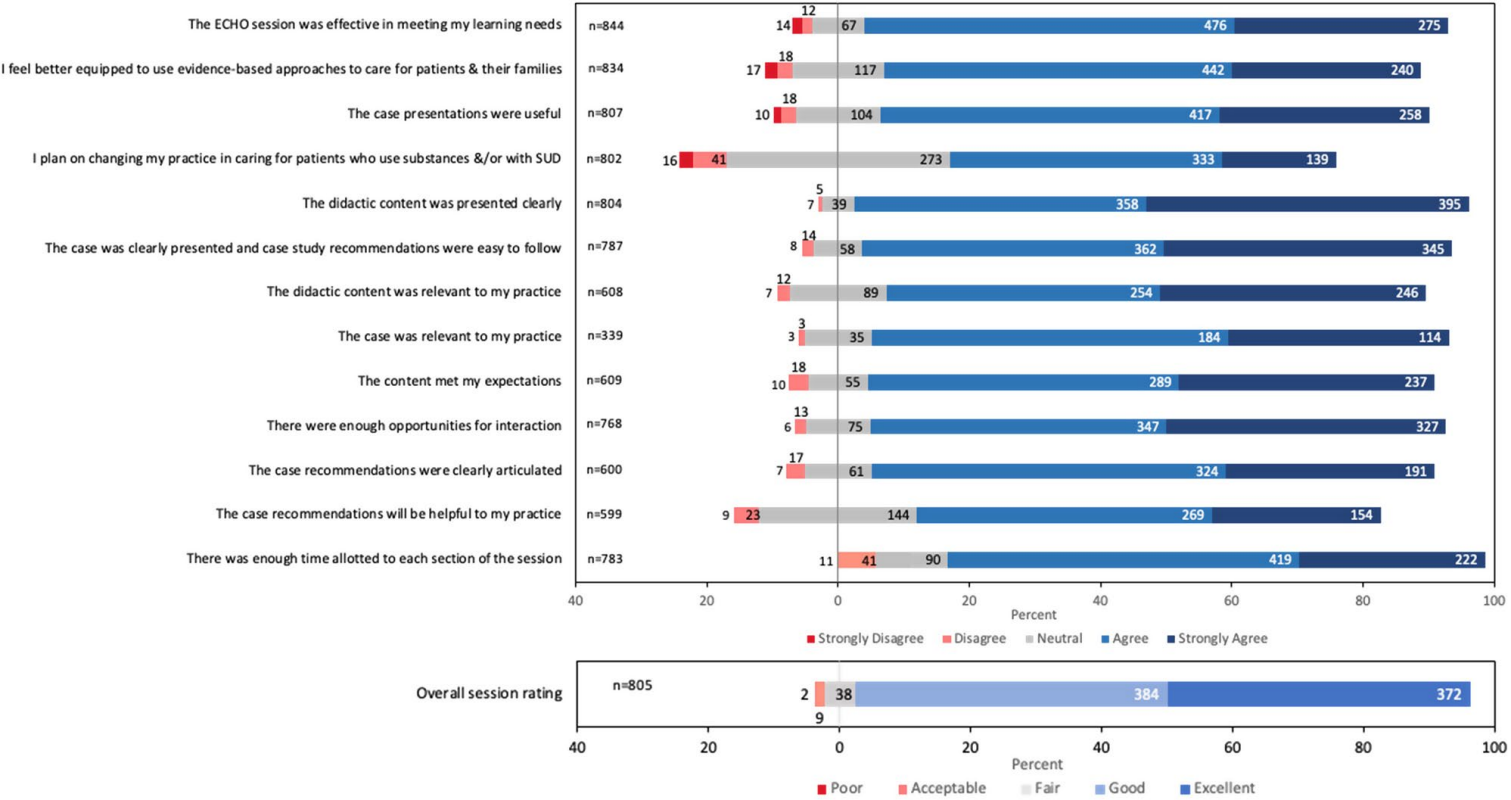
Participant session satisfaction ratings

See Fig. 2 for aggregated satisfaction ratings across post-session surveys. Of the 844 post-session evaluation surveys completed between June 2019 to July 2022, 93.9% rated overall program satisfaction as good or excellent. Mean ratings for content, format, interactivity, and session pace ranged from 4.0 to 4.4 out of 5 (SD 0.7 to 0.9). Accurate post-session survey participant demographics are not available because early cycles did not capture these data. Overall, sessions were perceived as effective at meeting learning needs (mean 4.2/5, SD 0.8), participants felt better equipped to use evidence-based approaches to care for patients and their families (mean 4.0, SD 0.8), and they planned to make practice changes because of what they learned (mean 3.7, SD 0.9).

Between January 2021 and June 2022, the 18 uploaded session recordings received 2,542 views (mean 73 times/session). Key themes from interviews relating to session content or format included that the sessions featured high quality speakers, good interactivity, and offered high quality learning unavailable elsewhere. For example, one participant noted, “*There’s no other place where I can get those types of presentations at that quality and at that level unless I was to take a higher-level university course*,* or*,* you know*,* if I actually worked in that type of profession*” (non-regulated service worker, AUD participant 11). One physician noted the value of the case discussions: “*Having the opportunity for people to share experiences…having time for conversation and for learning from each other is really important.”* (physician, OUD participant 24). Participants also indicated that the sessions offered practical and applicable supports. As highlighted by one participant, some indicated appreciating support with specific cases, but also more general advice and guidance for specific clinical scenarios that they could share with their teams and colleagues:

*I got out of it what felt like comprehensive support around managing withdrawal and managing risk factors and symptoms specifically related to withdrawal from alcohol and all symptoms. So being able to provide education around that within the scope of my role to the patients*,* and also to help support teams and around navigating that. So*,* it was just really*,* really clearly well laid out and presented and seemed supported*,* so that was excellent* (mental health and substance use team leader, AUD participant 12).

Participants also indicated an appreciation for learning from different perspectives and hearing from people outside of the city centres to support practical solutions for those working in more rural and remote settings.

Newsletters were reported to be beneficial for learning about upcoming sessions, provided credible information on useful topics, and were efficient for information sharing. As exemplified by one participant, the newsletters were available outside of the ECHO sessions for review and learning:

*I’m really thankful for that [newsletter on a pertinent topic] being there when I needed it*,* so I appreciate that you’re … always working on this topic…if there’s a question in substance use disorder*,* somebody is looking into it and some information about it will probably end up on here…I feel like this is a reliable reference* (pharmacist, AUD participant 16).

When participants were asked about what could be improved about the newsletter, they suggested having a summary of available topics for quick reference, and clinical information from recent sessions to pique interest or support recall. Listeners appreciated the podcast’s accessibility and the varied experiences highlighted, including lived/living experience perspectives from some guests.

### Objective 2: Knowledge, skills and supports for providers

Most podcast survey respondents reported increased knowledge about SUD care (84%) and confidence in SUD care (79%) after listening.

An important theme from the interviews centered around knowledge and skills gained, such as understanding available treatment options, managing treatment transitions, and gaining overall confidence in SUD care. Participants indicated using practical tips from the ECHO sessions to make treatment decisions and to support treatment transitions. This experience is exemplified in a quote from one participant about microdosing buprenorphine/naloxone:

*Because I had attended one of the ECHO sessions*,* I knew that 70 milligrams was completely reasonable to start doing the micro-dosing over to Suboxone [buprenorphine/naloxone] and so we saved that patient discomfort*,* and we did a really successful micro-dosing induction* (registered nurse, OUD participant 5).

Enhanced skills identified through interviews included screening patients and selecting appropriate treatment approaches, transitioning between treatments, and responding to precipitated withdrawal. Participants also reported greater knowledge and skills to prescribe new medications for patients, such as naltrexone, as well as making medication recommendations and providing guidance for patients based on patient-specific treatment goals. For example, one participant indicated,

*Increased knowledge around […] like all the different lines of treatment that are available*,* how to help screen and choose a treatment that’s going to be the best for the patient… learning how to transition a person from one treatment to another* (registered nurse, OUD participant 5).

Participants also indicated increased confidence to apply newly acquired skills. For example, one participant shared their journey at gaining confidence over time in managing different types of medications for opioid use disorder (OUD) and stated of the program:

*The impact of ECHO sessions on my ability to manage care for patients with OUD has been huge. Like – we were laughing about this the other day*,* […]my hand was shaking when I was writing my first Kadian [slow-release oral morphine] prescription*,* right? […] And I think just knowing there’s support out there to continue to expand the knowledge and*,* you know*,* other people that are like*,* “Yeah*,* you’re on the right track with this*,*” that’s been huge. So*,* ECHO’s been quite validating for the practice as well. […] a huge increase in confidence*,* for sure* (nurse practitioner, OUD participant 10).

Gaining confidence from peers and coming to terms with changing practice in the context of the pandemic was also critical, as described by one physician:

*How Project ECHO really helped me was navigating the uncertainty around being an OAT provider…going to all-virtual [care during] COVID. How are we supposed to do this work without seeing people in person?…that was terrifying… And then to see the people that I have trained with – and really respect their knowledge and commitment to this area – to see that they’re like: ‘Well*,* this is kind of what we’re doing. It’s okay. We’re all in this together.’ Nobody really knows…We obviously don’t have a clear picture of how to do it but if we proceed with documenting and doing our best*,* then that’s good enough. So I think that was the really helpful piece: This is how we’re going to move forward and this is just how it’s going to be*,* and I’m just going to have to get used to it.* (physician, OUD participant 26).

Participants also described how the ECHO platform supported shifting health providers’ attitudes, including reducing stigma towards people with SUDs, a critical component of providing ethical and quality care.

*This has been a stigma-based illness. It still is. It’s very*,* very difficult for people that are involved in caring for people to watch all of the diversity and inclusion work that goes on…you know*,* in our mental health and neurodiversity lens*,* we’ve made that a very inclusive environment*,* but unfortunately*,* the only people that are left on the outside now are people with substance use disorder…And that’s where I’m also planning to do [more work]… I’m starting to work on advocacy circles because it’s just too painful to watch people in our community. Like there’s one last group. Why would you leave one group out? …you have to make a personal decision…if you’re going to be an ally*,* then you know*,* in allyship you have to stand forward. So I’m increasingly moving forward and I feel very encouraged with what I’m seeing*,* and BC ECHO has been probably the biggest sign of hope.* (senior clinician consultant, OUD participant 7).

One provider explained that the facilitated discussion, which fostered open sharing, was a key factor in supporting this shift for clinicians:

*I think [the BC ECHO on Substance Use is] just a really good opportunity to have unbiased conversations with colleagues who are dealing with the same kind of stuff. Often in OAT and OUD treatment we come across those cases that are… I don’t want to say unethical*,* but there are a lot of factors that could cause health care professionals to be unsure of how to continue treatment*,* especially when there are other medications or other co-occurring disorders happening. So I think that ECHO is a pretty unbiased platform for people to share the information*,* especially if you have the right person moderating.* (mental health clinician, OUD participant 19).

### Objective 3: Participant-reported changes to practice, programs or policy

Of podcast survey respondents, 67% intended to make practice changes based on episode content. Interview participants indicated that as a result of participating in ECHO, they were better equipped to facilitate shared decision-making with patients regarding treatments and care planning. For example, one participant stated,

*I had a client that I was working with … and after taking the pharmacotherapy lecture*,* we started talking about medication … So*,* then we were able to have a conversation about other options and he was able to take this knowledge … and he ended up trying a different medication*,* and that was successful for him* (nurse practitioner, OUD participant 3).

Examples of evidence-informed practice changes implemented by interview participants as a result of the OUD and AUD ECHO sessions included changes in prescribing practices (e.g., types of medication, dosages, transitioning patients to different pharmacotherapy options) and better managing precipitated withdrawal and buprenorphine inductions. One participant mentioned that after attending the AUD ECHO sessions, they felt like they were able to *“…more confidently have conversations about benefits and disadvantages to different types of medications*,* first line treatment options versus other treatment options…”* (Counsellor, AUD participant 3).

Some participants also reported changes in their treatment approach for patients with substance use disorder, utilizing a harm reduction approach and trauma-informed lens to communicate with their patients in a non-judgmental and compassionate manner. As noted by one participant,

*I really think my compassion for people has increased. My understanding of the nature of trauma in all substance use disorders definitely has changed my practice and my approach with people … we can’t just look after one part of their being; we must look after all parts of their being…* (registered nurse, AUD participant 24).

In addition, participants spoke about consolidating learning from ECHO and applying it to practice. Specifically, participants indicated applying new learning about clinical assessments, diagnostic testing for patients with AUD and OUD, such as using standardized screening tools, and changing their use and interpretation of urine drug screening. For example, participants indicated greater understanding regarding the time frames of substances appearing in urine and omitting drug testing in circumstances when it is not that helpful. One participant described applying knowledge learned about drug screening considerations by presenting his own clinical case for discussion,

*[an example is]…urine drug screens*,* and what’s the right way to do those*,* and all the considerations around what’s going to show up and what’s not going to show up. [The session] just helps to solidify our practice and knowledge. Again*,* I had a really challenging case and was able to have my situation presented*,* and get input and feedback and*,* yeah*,* I think all of [the sessions] are applicable to what I’m doing*,* so they’ve been very good* (nurse practitioner, OUD participant 10).

### Objective 4: Fostering a community of practice

Interview participants reported that sharing and translating the knowledge and skills from the ECHO sessions with other health care providers, allowed them to expand their networks. For example, one participant noted:

*Oh [my clinical network of OUD providers] strengthened and expanded … not only has it strengthened mine and broadened my scope but I’m able to share that and broaden others that I work with”* (social worker, OUD participant 4). The sense of community that developed among participants was reported to contribute to feeling supported and reducing professional isolation, exemplified by one participant:

*I think often we think we’re alone*,* or we’re the only one who’s going through this*,* so I think some of the cases […] more than one person has kind of had to deal with or come across. […] I know now if I had a certain case that I had to deal with like outside*,* that someone else has dealt with*,* it’s a lot easier to be like*,* ‘Oh*,* hey*,* remember this? This clinic dealt with it this way*,* and maybe we can give them a call’…* (registered nurse, OUD participant 13).

## Discussion

This article describes a set of innovations to augment the standard ECHO model to enhance reach, participant satisfaction, and impact for interprofessional office-based SUD care providers working in primary care and substance use treatment settings. In keeping with previous research, program participants reported that the program effectively built knowledge, confidence, and capacity in delivering SUD care. This work is particularly critical in the context of the escalating toxic drug poisoning crisis and identified need for rapid upskilling of interdisciplinary health care professionals to provide evidence-based SUD care.

### Reach

The steady growth of the community of practice demonstrates ongoing program interest and engagement, facilitated by open and rolling registration. We were successful at reaching primary care providers, and clinicians in rural settings were overrepresented (36% of registrants, vs. 13% of BC’s population is rural) [16]. The proportion of registrants identifying as female was congruent with provincial health care workforce representation (78% vs. 80%) [17].

Despite this positive reach to rural settings, challenges remained in engaging health care providers from Yukon Territory. Feedback following an early debrief session with the Steering Committee indicated challenges in identifying providers doing addiction work in Yukon to target recruitment and promotion activities, as well as concerns about the complexity of the cases presented. These discussions highlighted system-level challenges and support the consideration of more foundational topics that could reach providers early on in their SUD care practice.

### Outcomes and impact

As in other published studies, our program evaluation demonstrates that the ECHO model is a feasible and acceptable way to deliver training and education on SUD to a range of interdisciplinary service providers [18]. Most participants rated their satisfaction with the sessions as ‘good’ or ‘excellent’ and planned on making practice changes because of their participation in the program, while interview findings highlight key features that resonated with these participants.

The reported increases in knowledge of SUD clinical management and confidence to discuss treatment options with patients is aligned with other published evaluation findings of substance use focused ECHO programs [13, 19, 20]. Like other studies, participants found session content relevant, and intended to apply learnings to practice [18]. Our qualitative findings detailed specific clinical skills and knowledge that participants intended to apply, such as the appropriate use of urine drug screening, evidence-based screening and assessment approaches, and managing transitions between pharmacotherapies. These applications involve clinical reasoning and support best practices, which reaffirmed the goals of the program.

Stigma and misperceptions about OUD and reticence to prescribe OAT have been cited elsewhere as key barriers to providing OUD care among ECHO participants [21]. Respondents reported greater awareness of how to embed principles of trauma-informed care and a philosophy of harm reduction in their practice, as well as decreased stigma, which can improve patient engagement in care. Having these approaches modelled through the discussion of real patient cases provided a blueprint for how to adopt these approaches in practice. Enhanced self-awareness in patient interactions has been cited as a positive outcome of attending ECHO sessions and one possible reason for this shift in attitudes [22].

The development of clinical treatment networks and the sharing of newly acquired knowledge and skills with colleagues supports the sustainability of the initiative. Reduced professional isolation is a priority of the ECHO model in general and has been cited as a positive outcome of an OUD-focused ECHO program [12, 20]. This connectivity is even more critical in the rapidly evolving SUD care field where relatively few trained providers exist for ongoing mentorship.

### Benefits of ECHO model adaptations

#### Enhancing reach

A common criticism of urban-centric programs is their failure to reflect the realities of rural settings adequately (e.g., fewer services, providers, resources); as a result, they are less appealing to rural learners. In response, we intentionally engaged a diverse, regionally representative hub team and believe it contributed to greater uptake outside urban centres. Some regions had lower uptake; targeted promotion could increase reach to these underrepresented areas.

#### Improving accessibility

Consistent high attendance indicates that short, frequent sessions (1 h, bi-weekly) were acceptable. Previous investigations of OUD-focused ECHO programs have indicated time and clinical commitments as key barriers to participation and that holding shorter sessions over the lunch hour seem to be successful at encouraging participation [18]. In addition to reaching primary care providers, the programs had consistent representation of nursing and other allied health disciplines, indicating that a great need for education and training on SUD care among diverse health professional disciplines remains. Competing demands have been cited as barriers to engagement [13]; however our short live sessions and online archived presentations (suggestions from other authors) [12]), enabled participation both on- and offline. Early feedback from a large rural region indicated that cases were quite complex and advanced for many learners. In response, we mixed in simplified composite cases that removed some complexities to improve accessibility and to focus learning on core clinical knowledge and skills.

#### Balancing consistency with nimbleness

Our didactic curriculum was developed in-house by a team of medical writers with experience in developing provincial clinical guidelines and online education. This adaptation led to consistency in presentation style and format, clear and attainable learning objectives for each presentation and a curriculum that was based on evidence-based clinical guidelines and recommendations. Many other ECHO programs rely on subject matter experts to develop their presentations in isolation. This approach would not have been feasible given the limited capacity of substance use experts managing the demands of the overdose crisis. Our streamlined approach ensured didactic presentations followed a similar format and covered the most current evidence, while also reducing the burden on individual presenters to create content for the series. This approach also facilitated rapid adaptation when needed. In the context of the early months of the COVID-19 pandemic, as new clinical guidance was emerging, the ECHO team was able to adapt and deliver two unique sessions during time already in participants’ calendars. In this way, the team was able to use an existing approach to reach a large number of clinicians and provide updated guidance rapidly. Several other ECHO programs have employed this approach in the context of the emergency response to the COVID-19 pandemic – either by using an existing ECHO network to disseminate information about rapidly changing clinical best practices, or by re-tasking existing ECHO programs to deliver COVID-19 content [14–17]. 

#### Supplementing session content

The novel program delivery formats, in addition to regularly scheduled ECHO sessions (i.e., podcast, blog posts, newsletter) to improve accessibility and uptake were well received. Clinical resources were downloadable and supported access when attendance at virtual ECHO sessions was not possible. Asynchronous podcasts facilitated access to content any time, as standalone resources, and provided an additional format for knowledge acquisition, attending to different learning styles. The continued growth in podcast downloads, and the listening length of 75%+ of the episodes demonstrated their relevance and engagement of new audiences. Newsletters facilitated ongoing communication with registrants, regardless of session attendance. Increased marketing could help to augment their reach and impact.

### Limitations and challenges of ECHO model innovations

An overall dearth of experienced clinicians in SUD care exists in BC and Yukon to engage as ‘expert’ didactic presenters. Those versed in addiction medicine are often stretched across clinical, leadership, administrative and/or research roles, limiting their available time for participation.

Recruiting clinicians to submit and present clinical cases was sometimes challenging. Cases that were submitted reflected real-life complexities, often including intersections of several types of substance use/SUD, mental health issues, housing challenges and other social issues. In response, some simplified cases were developed to reflect real but pared down or composite clinical scenarios. These helped to reinforce core learning concepts with moderate complexity and could be used when no real case was submitted.

We prioritized regional diversity in presenters and hub team members, yielding a small and varied hub team at each session (e.g., 2–3 clinical experts/session). In future, we would strive for a larger hub team and a funding model that supports regular attendance and participation through compensation.

Relatively low evaluation response rates present the potential for response bias by engaged participants. However, despite these rates, it is reasonable to infer from repeat attendances that participants found the sessions valuable. Lack of awareness of newsletters and podcasts by many interview participants suggests more promotion is needed. While the 53 interviews yielded practical examples of practice changes and greater depth of understanding of participants’ satisfaction with the program relative to their needs, these data may not reflect the experiences of all ECHO participants and should be interpreted accordingly.

## Conclusions

Our program evaluation findings build on existing literature that suggests that Project ECHO is a feasible and acceptable education model for upskilling prescribers and other health care providers in the clinical management of OUD and AUD. Participants reported increased knowledge, skills and intentions to make changes in their clinical practice. Participants also reported attitude changes, including more confidence in incorporating harm reduction and trauma informed approaches. Forming connections to supportive clinical networks was another key benefit of program participation, especially for rural providers who described isolation as a barrier to ongoing support and mentorship. Novel formats (i.e., podcast, blog posts, newsletters), rolling registration and short lunch sessions, are adaptations that likely contributed to enhanced reach and accessibility across a large and diverse geographic area. Further, drawing on a diverse and varied hub team, and using a centralized didactic presentation development process supported the involvement of crucial experts despite their limited capacity to provide ongoing mentorship in the context of the overdose crisis. These benefits may be particularly pertinent in fields like substance use, where a large knowledge-to-practice gap exists and there are few established resources for mentorship. Archived content, biweekly (less frequent) sessions, and in-house content development reduced burden on both participants and hub team members in the context of the overdose crisis that stretched clinicians. These adaptations may be considered by other ECHO programs to meet their local contexts and needs.

## Electronic supplementary material

Below is the link to the electronic supplementary material.

Supplementary Material 1

Supplementary Material 2

Supplementary Material 3

## Data Availability

The quantitative datasets used and/or analyzed during the current study are available from the corresponding author on reasonable request.

## Abbreviations

SUD: Substance use disorders
ECHO: Extension for Community Healthcare Outcomes
BC: British Columbia
US: United States of America
OUD: Opioid use disorder
AUD: Alcohol use disorder
BCCSU: British Columbia Centre on Substance Use
OAT: Opioid agonist treatment
SROM: Slow-release oral morphine
COVID-19: Coronavirus disease of 2019; the name of the disease caused by the SARS-CoV2 virus
