# Supplementary material for: Innovations to the ECHO model to enhance reach and network-building among addiction clinicians in Western Canada
Source: Addict Sci Clin Pract. 2024 Dec 18;19:93. doi: 10.1186/s13722-024-00524-z (PMC11654416; doi:10.1186/s13722-024-00524-z)
Supplement: Supplementary file 1 — Supplementary Material 1 [file 13722_2024_524_MOESM1_ESM.docx]

**Additional file 1: Topics and release dates for ECHO sessions, clinical tools, podcasts and blog posts**

**OPIOID USE DISORDER (OUD) ECHO SESSIONS (39 sessions)**

| **Session #** | **Date** | **ECHO Session Topic** |
| --- | --- | --- |
| **Cycle 1**  **1** | Jun 27, 2019 | Caring for patients with an OUD in your primary care practice setting: An overview |
| **2** | Jul 11, 2019 | Decoding urine drug tests |
| **3** | Jul 25, 2019 | Practical considerations for buprenorphine/naloxone induction in primary care settings (traditional and home induction): Part 1 (includes micro-dosing inductions) |
| **4** | Aug 8, 2019 | Practical considerations for buprenorphine/naloxone induction in primary care settings (traditional & home induction): Part 2 |
| **5** | Aug 22, 2019 | Opioid agonist treatment (OAT) and pharmacy |
| **6** | Sep 12, 2019 | Transitions between pharmacotherapies for OUD: Part 1 (Methadone focus) |
| **7** | Sep 26, 2019 | Transitions between pharmacotherapies for OUD: Part 2 (Slow-release oral morphine [SROM] focus) |
| **8** | Oct 10, 2019 | Managing patients with co-occurring chronic pain and OUD |
| **9** | Oct 24, 2019 | Continuity of care when transitioning between care setting (i.e., between acute and primary settings, pre- and post-incarceration) |
| **10** | Nov 14, 2019 | Special considerations for youth and pregnancy |
| **Cycle 2**  **1** | Feb 4, 2020 | Caring for patients with an OUD in your primary care practice setting: An overview |
| **2** | Feb 20, 2020 | OAT and pharmacy |
| **3** | Mar 5, 2020 | Managing patients with co-occurring chronic pain and OUD |
| **4** | Mar 25, 2020 | Caring for patients on OAT during the COVID-19 pandemic: Part 1 |
| **5** | Apr 7, 2020 | Caring for patients on OAT during the COVID-19 pandemic: Part 2 |
| **6** | Apr 21, 2020 | Special considerations for OUD in pregnancy |
| **7** | May 5, 2020 | Practical considerations for buprenorphine/naloxone induction in primary care settings: Precipitated withdrawal & home induction |
| **8** | May 21, 2020 | Practical considerations for the clinical management of AUD in the context of COVID-19 |
| **9** | Jun 9, 2020 | Transitions between pharmacotherapies for OUD (methadone to buprenorphine/naloxone) |
| **10** | Jun 23, 2020 | Special considerations for OUD in youth |
| **Cycle 3**  **1** | Jul 21, 2020 | Decoding urine drug tests |
| **2** | Aug 6, 2020 | Caring for patients with OUD in your primary care practice setting: An overview |
| **3** | Aug 25, 2020 | OAT and pharmacy |
| **4** | Sep 10, 2020 | Extended-release buprenorphine |
| **5** | Sep 24, 2020 | Managing patients with concurrent mental health and OUD |
| **6** | Oct 8, 2020 | Practical considerations for buprenorphine/naloxone induction in primary care settings (home induction) |
| **7** | Oct 22, 2020 | Special considerations for OUD in pregnancy |
| **8** | Nov 17, 2020 | Transitions between pharmacotherapies for OUD (SROM focus) |
| **9** | Dec 1, 2020 | Continuity of OUD care across care settings: From corrections to community |
| **10** | Dec 15, 2020 | Continuity of OUD care across care settings: Acute to community |
| **Cycle 4**  **1** | Feb 11, 2021 | Decoding urine drug tests |
| **2** | Feb 25, 2021 | Extended-release buprenorphine |
| **3** | Mar 11, 2021 | OAT and pharmacy |
| **4** | Mar 25, 2021 | Transitions between pharmacotherapies for OUD (methadone to buprenorphine/naloxone) |
| ***5** | Apr 13, 2021 | Managing co-occurring AUD & OUD (*co-delivered as AUD Cycle 2 Session 4) |
| **6** | Apr 29, 2021 | Managing patients with co-occurring chronic pain and OUD |
| **7** | May 18, 2021 | Managing patients with co-occurring opioid and stimulant use disorders |
| **Cycle 5**  **1** | Apr 28, 2022 | OUD and primary care practice settings |
| **2** | Jun 23, 2022 | Ordering and interpreting urine drug tests for OAT |

Note: OUD=opioid use disorder; OAT=opioid agonist treatment; SROM=slow-release oral morphine; AUD=alcohol use disorder

**ALCOHOL USE DISORDER (AUD) ECHO SESSIONS (14 sessions)**

| **Session** | **Date** | **ECHO Session Topics** |
| --- | --- | --- |
| **Cycle 1**  **1** | Aug 18, 2020 | Introduction to high-risk drinking and AUD in British Columbia |
| **2** | Sep 15, 2020 | Screening for high-risk drinking and AUD |
| **3** | Oct 1, 2020 | Pharmacotherapy approaches for AUD |
| **4** | Nov 3, 2020 | Alcohol withdrawal management |
| **5** | Nov 26, 2020 | Brief intervention for high-risk drinking and AUD in primary care |
| **6** | Dec 8, 2020 | Psychosocial and community-based interventions for AUD |
| **Cycle 2**  **1** | Jan 19, 2021 | Considerations for AUD care across the age spectrum |
| **2** | Feb 2, 2021 | Harm reduction approaches to high-risk drinking and AUD |
| **3** | Mar 2, 2021 | Considerations for high-risk drinking or AUD in pregnancy |
| ***4** | Apr 13, 2021 | Managing co-occurring AUD & OUD (*co-delivered as OUD Cycle 4 Session 5) |
| **5** | May 11, 2021 | Pharmacotherapy approaches for alcohol use disorder |
| **6** | Jun 1, 2021 | Alcohol withdrawal management |
| **Cycle 3**  **1** | May 31, 2022 | AUD and chronic pain |
| **2** | Jul 21, 2022 | Screening and brief intervention for AUD |

Note: AUD=alcohol use disorder; OUD=opioid use disorder

**CLINICAL TOOLS (2)**

| **Type of resource** | **Date** | **Clinical Tool Title** |
| --- | --- | --- |
| **Frequently Asked Questions** | Mar 24, 2020 | [Caring for patients on OAT during the COVID-19 pandemic: Part 1](https://www.bccsu.ca/faq-caring-for-patients-on-oat-during-the-covid-19-pandemic/) |
| **Frequently Asked Questions** | Apr 7, 2020 | [Caring for patients on OAT during the COVID-19 pandemic: Part 2](https://www.bccsu.ca/faq-caring-for-patients-on-oat-during-the-covid-19-pandemic-part-2/) |

Note: OAT=opioid agonist treatment; COVID-19=coronavirus disease 2019

**PODCAST:** [**Addiction Practice Pod**](https://bcechoonsubstanceuse.ca/podcast/) **(12 episodes)**

| **Episode** | **Release Date** | **Podcast Topic** |
| --- | --- | --- |
| **Season 1**  **1** | Sep 22, 2020 | Supporting patients with substance use disorders to achieve their recovery goals |
| **2** | Oct 6, 2020 | Bringing substance use care into your primary care practice |
| **3** | Oct 20, 2020 | Substance use disorders and trauma- and violence-informed care |
| **4** | Nov 4, 2020 | Providing compassionate care to young people who use substances |
| **5** | Nov 24, 2020 | The harms of criminalizing substance use |
| **6** | Dec 1, 2020 | Indigenous perspectives on health and wellness, substance use, and harm reduction |
| **Season 2**  **1** | Oct 29, 2021 | Substance use in rural and remote contexts |
| **2** | Nov 12, 2021 | Treatment and care for AUD |
| **3** | Nov 26, 2021 | Treatment considerations for co-occurring substance use |
| **4** | Jan 14, 2022 | Co-occurring pain and OUD |
| **5** | Jan 28, 2022 | Psychosocial treatment approaches |
| **6** | Feb 11, 2022 | Improving substance use care for racialized groups |

Note: AUD=alcohol use disorder; OUD=opioid use disorder

[**BLOG**](https://www.bccsu.ca/blog/) **(9 posts)**

| **Publication Date** | | **Blog Post Topics** |
| --- | --- | --- |
| **1** | Jan 10, 2020 | Into Practice: Ivana Gojkovic, Coordinator, Clinical and Distribution Pharmacy Services at Provincial Health Services Authority |
| **2** | Jan 21, 2020 | Presenter spotlight: Kristine Rowswell, Family Nurse Practitioner, HIV/HCV/OAT Specialized Support Team, Northern Health |
| **3** | Mar 1, 2020 | Into Practice: Ann Johnston, Manager, Pharmacy Practice Support at the BC Pharmacy Association |
| **4** | Nov 26, 2020 | The harms of criminalizing substance use: An interview with Dr. Bonnie Henry |
| **5** | Feb 24, 2021 | Changing the story on addiction care |
| **6** | Apr 23, 2021 | Pharmacists at the forefront |
| **7** | Jun 24, 2021 | Seeing beyond the behaviour |
| **8** | Jul 16, 2021 | Mentorship in medicine |
| **9** | Aug 13, 2021 | Alcohol harm reduction |

Note: HIV=human immunodeficiency virus; HCV=hepatitis C virus; OAT=opioid agonist treatment
