# Supplementary material for: Innovations to the ECHO model to enhance reach and network-building among addiction clinicians in Western Canada
Source: Addict Sci Clin Pract. 2024 Dec 18;19:93. doi: 10.1186/s13722-024-00524-z (PMC11654416; doi:10.1186/s13722-024-00524-z)
Supplement: Supplementary file 2 — Supplementary Material 2 [file 13722_2024_524_MOESM2_ESM.docx]

**BC ECHO on Substance Use Pre-Session (Registration) Survey**

1. **Name:** _________________
2. **Email address:** __________________
3. **I have previously attended BC ECHO on Substance Use sessions**

- Yes
- No

1. **What do you hope to gain by attending the upcoming ECHO session? Check all that apply:**

- I am interested in networking/connecting with other practitioners and addiction specialists across BC and the Yukon
- I am interested in increasing my knowledge and skills related to evidence-based practice for substance use care
- I would like to discuss and contribute to recommendations on a case study
- I am looking for clinical guidance regarding questions that have come up in my own practice/work related to substance care
- I am interested in obtaining a CME credit

**BC ECHO on Substance Use Post-Session Survey**

**1. Please indicate your agreement with the following statements:**

*Likert scale: Strongly Disagree = 1; Disagree = 2; Neutral = 3; Agree = 4; Strongly Agree = 5*

1. The ECHO session was effective in meeting my learning needs
2. As a result of participating in the ECHO session, I feel better equipped to use evidence-based approaches to care for patients and their families in my practice"
3. The case presentation was useful
4. As result of having at tended this ECHO session, I plan on changing my practice, related to the provision of care to patients who use opioids and/or those with OUD

**2. As of result of having attended this ECHO session, I plan on changing my practice is the following ways:**

*[Open text]*

**3. Can you identify any barriers to incorporating what you learned today into your practice?** **Select all that apply:**

- *Insufficient knowledge or experience*
- *No opportunity to implement intervention*
- *Organizational barriers*
- *Financial barriers*
- *No barriers/no plans for change*
- *Other (please specify): ___________*

**4. Please indicate your agreement with the following statements:**

*Likert scale: Strongly Disagree = 1; Disagree = 2; Neutral = 3; Agree = 4; Strongly Agree = 5*

**Format**

1. The session lead communicated effectively during the didactic presentation
2. The case was clearly presented and case study recommendations were easy to follow

**Content**

1. The content presented in the didactic presentation was relevant to my practice
2. The content presented in the case was relevant to my practice
3. The content presented met my expectations
4. The case recommendations that were developed and distributed were clearly articulated
5. The case recommendations that were developed and distributed will be helpful to my practice

**Interactivity**

1. There were adequate opportunities for interaction (e.g. questions)

**Pace**

1. There was an appropriate amount of time allotted to each section of the ECHO session

**5. Please rate the overall ECHO session:**

*Poor = 1; Acceptable = 2; Fair = 3; Good = 4; Excellent = 5*

**Continuing Medical Education Accreditation Questions**

| **6. Did you perceive any bias, whether industry, or other?**   - *Yes* - *No* - *Unsure* | | | | |  |  |  |  |  |  |
| --- | --- | --- | --- | --- | --- | --- | --- | --- | --- | --- |
| **7. Were the stated learning objectives met?**   - *Yes* - *No* - *Unsure* | | | | |  |  |  |  |  |  |
| **8. Did the presentation offer balanced views and recommendations across all relevant options related to the session topic?**   - *Yes* - *No* - *Unsure*   **Recommendations** | | | | | | |  |  |  |  |
| **9. Additional comments or suggestions?**  *[Open text]* |  |  |  |  |  |  |  |  |  |  |

**BC ECHO on Substance Use Podcast Survey**

**Knowledge Check Questions for each episode:**

Response options: *True/False/Do not know*

| **Episode** | **Questions** |
| --- | --- |
| **1** | - 1. Risk of overdose is highest for those who have just left recovery centres. *[True]*   2. Initiation of opioid agonist therapy before discharge is likely to prevent outpatient relapses. *[True]*   3. Medically supervised withdrawal is sufficient to produce long-term recovery. *[False]*   4. The majority of residential treatment programs in British Columbia offer opioid agonist therapy. *[True]* |
| **2** | - 1. Opioid Agonist Treatment can reduce the risk of overdose among people who use drugs. *[True]*   2. There are specific Medical Service Plan (MSP) [provincial health care coverage] codes for family doctors in BC that support family doctors to provide care for substance use disorders to their patients. *[True]*   3. Consulting with people with lived and living experience can improve a primary care practitioner’s practice. *[True]*   4. It is difficult to incorporate opioid agonist treatment into an existing primary care practice. *[False]* |
| **3** | 1. You must be a mental health practitioner to be able to implement trauma-informed care in your practice. *[False]* 2. Trauma manifests itself differently in different people, and can include physical symptoms, like trouble sleeping, irritability, or pain. *[True]* 3. Self-reflection is an important part of clinical care. *[True]* 4. Being compassionate and practicing acceptance of patients during clinic visits creates a safer space for both patient and practitioner. *[True]* |
| **4** | 1. When it’s appropriate, and with the permission of the young patient, you can involve supportive family members in a patient’s care. *[True]* 2. Primary care practitioners can help reduce harms in their young patients’ lives by recommending community resources and helping facilitate access to them. *[True]* 3. Treatment approaches for youth with substance use disorders are the same as those for adults. *[False]* 4. The BCCSU’s youth supplement to the Clinical Guideline on Opioid Use Disorder Management discusses some of the subtleties involved in treating opioid use disorder in young people. *[True]* |
| **5** | 1. Substance use disorder is a criminal justice issue, not a public health issue. *[False]* 2. As clinicians, we have powerful voices to advocate for change to systemic issues that create harms for our patients. *[True]* 3. A 2019 report by the current Provincial Health Officer of British Columbia Dr. Bonnie Henry outlines the many health harms created by the criminalization of people who use drugs. *[True]* 4. When people are released from incarceration, risk of overdose death, HIV and Hep C decrease. *[False]* |

**2. Do you anticipate making any changes to your clinical practice as a result of the episode you listened to?**

- *Yes*
- *No*
- *Unsure*

**3. What changes do you anticipate making to your clinical practice?**

| **Episode** | **Response options (Please choose all that apply):** |
| --- | --- |
| **1** | - Referring patients to recovery facilities that offer opioid agonist treatment - Prescribing opioid agonist treatment before inpatient discharge from a recovery centre - Supporting long term recovery by pairing medically supervised withdrawal with other treatment - Other (please specify): _____________________________ |
| **2** | - - - Undertaking further education in providing opioid agonist treatments, e.g., Provincial Opioid Addiction Treatment Support Program (POATSP) [online learning modules], BC ECHO on Substance Use     - Incorporating the provision of opioid agonist treatments into my practice - Implementing motivational interviewing in my practice   - - Improving my knowledge of screening tools for substance use disorders - Consulting with people with lived experience to improve my practice - Other (please specify): __________________________ |
| **3** | - Altering the physical set-up of my clinic - Working to increase my self-reflection at work - Implement trauma- and violence-informed care in my practice - Seek further training on trauma- and violence-informed care - Working to practice more compassion, for my colleagues and for myself - Other (please specify): _______________ |
| **4** | - - - Changing my communication style and approach with young patients     - Considering involving supportive family in care plans for my patients, if appropriate and with permission     - Improving my knowledge of community resources and supports for youth - Other (please specify): ________________________ |
| **5** | - - - Improving my awareness of the health harms of the criminalization of drug use     - Using my voice to advocate for addressing systemic issues affecting my patients     - Other (please specify): _______________ |

**4. Did listening to this episode increase your knowledge about substance use disorder care?**

- *Yes*
- *No*
- *Not applicable*

**5. Did listening to this episode increase your confidence in managing relevant cases?**

- *Yes*
- *No*
- *Not applicable*

**6. Did this episode help you in any other way?**

*[Open text]*

**7. Is there any way that this podcast could be improved?**

*[Open text]*
