# Supplementary material for: Innovations to the ECHO model to enhance reach and network-building among addiction clinicians in Western Canada
Source: Addict Sci Clin Pract. 2024 Dec 18;19:93. doi: 10.1186/s13722-024-00524-z (PMC11654416; doi:10.1186/s13722-024-00524-z)
Supplement: Supplementary file 3 — Supplementary Material 3 [file 13722_2024_524_MOESM3_ESM.docx]

**Evaluation of Opioid Use Disorder (OUD) Project ECHO**

**OUD QUALITATIVE INTERVIEW COVER SHEET**

**COVER SHEET FOR PARTICIPANTS REGARDING THEIR EXPERIENCE WITH OPIOID USE DISORDER (OUD) PROJECT ECHO**

**Interviewer: ____________________________________________________________**

**Date and Time: _________________________________________________________**

**File Name: _____________________________________________________________**

**DEMOGRAPHICS – DO NOT TURN ON AUDIO RECORDER**

**1. Do you identify your gender as:**

Man Woman Intersex Transgender  Non-Binary

Two-spirit Prefer not to say Prefer to self-describe: ________________________

**2. How old are you? ____________________**

**3. What do you identify your race or ethnicity as?**

Indigenous / Aboriginal White (Caucasian) South Asian

East or Southeast Asian Black / African Canadian    Other: _______________

**4. What is the highest level of education you received?**

Trade / technical training  Bachelor’s Degree  Professional Degree

Master’s Degree        Doctorate Degree Other: ___________________________

**5. What is your occupation?**

Clinician Physician Pharmacist       Social Worker

Registered Nurse (RN)             Nurse Practitioners Other: ____________________________

**6. (If participant’s occupation is physician) what is your training background?**

Family medicine Internal medicine Psychiatry

Emergency medicine Preventative Medicine  Other: _______________________

**7. In which health region are you currently working?**

Vancouver Coastal Health Authority Fraser Health Authority

Interior Health Authority Vancouver Island Health Authority

Northern Health Authority First Nations Health Authority

Yukon Territory Health Region     Provincial Health Services Authority

**8. What kind of the health care setting are you currently working?**

Emergency care Acute care Community care Other: _______________________

**9. How would you describe the geographic location of your place of work?**

Urban Rural Neither Other: _____________________

**10. Have you completed any non-degree substance-use related education/training?**

Yes Please explain: __________________________________

No

**11. Have you completed any degree training in addiction medicine? (e.g. postdoctoral fellowship)**

Yes Please explain: ___________________________________

No

**12. If you are a prescriber, how many years of experience do you have prescribing opioid agonist treatment?**

< 1 year 1-2 years 3-4 years 5-10 years 10-20 years 20-30 years 30-40 years

**13. If you are a non-prescriber, how many years of experience do you have managing and treating patients with opioid use disorder?**

< 1 year 1-2 years 3-4 years 5-10 years 10-20 years 20-30 years 30-40 years

**14. What is the approximate date of the first ECHO session you participated in?** _(dd/mm/yyyy)____________

**15. When is the approximate date of the last ECHO session you participated in?** _(dd/mm/yyyy)____________

**OUD Project ECHO Interview Guideline**

**OPIOID USE DISORDER (OUD) PROJECT ECHO (EXTENSION FOR COMMUNITY HEALTHCARE OUTCOMES) PARTICIPANT INTERVIEW GUIDE**

**DIRECTIONS**

Thank you for agreeing to share your experience as a health care provider who has experience providing treatment and managing patients with opioid use disorder (OUD) in British Columbia or the Yukon. Today, you will be taking part in an approximately 45-minute interview about your involvement in the OUD Project ECHO. We will ask questions about your experience as a participant of the OUD Project ECHO, as well as some questions about your past educational experiences and how these experiences have informed your work as a health care provider. With your permission, this interview will be audio-recorded and later transcribed by a professional transcriptionist. Before we begin, please note that everything that you share with us is confidential, and that your information or identity will not be shared with anyone outside of our project team, unless required by law. Given your role as a health care provider, you will be assigned a pseudonym and any potentially identifying details will be anonymized to protect your identity. Also, please keep in mind that there are no right or wrong answers to these questions, and you do not have to answer any questions that you are not comfortable answering.

|  | |
| --- | --- |
| **SECTION 1: PREVIOUS TRAINING AND EDUCATION OF OUD—PRIOR TO PARTICIPATION IN OUD PROJECT ECHO** | |
|  | |
| **“We are interested in learning about your previous training and education of opioid use disorder prior to your participation in OUD Project ECHO, as well as any limitations you had in your previous training when preparing you to work with patients with opioid use disorder…”** | |
| ***QUESTIONS*** | ***PROBES*** |
| What motivated your decision to work with people who use substances? | - *What previous experiences have you had working with people who use substances?* - *Did these past experiences play a role in your decision to enter this area of practice? If so, how?* |
| What training(s) or education have you received related to OUD?    For example: Provincial Opioid Addiction Treatment Support Program, Addiction Care and Treatment Online Course, attended a seminar on OUD clinical guidance delivered by BCCSU or completed a postdoctoral fellowship in Addiction Medicine | - *What type of training was it?* - *What type of advanced education was it?* - *How long ago was it?* - *Was the training in-person or virtual?* - *If it was* ***in-person****, where was it located?* - *Do you have a preference between in-person and virtual training/advanced education?* - *What were your impressions of the training?* - *What could have been done to improve the training or advanced education?* |
| Did the training adequately prepare you to provide care to people who use substances? | - *Why/why not?* - *What were you prepared for? What were you not prepared for?* - *Do you think this training would be considered up to date with current guidelines and recommendations on OUD?* |
| What could have been done to improve your training / education? | - *What kind of training do you think would be beneficial for health care providers?* |
| Prior to participation in OUD Project ECHO, what was your source of learning or information related to OUD? | - *Did you use uptodate?* - *Did you use primary literature?* - *Did you use evidence-based resources, Canadian resources, or BCCSU specific resources for your learning?* - *Did you gather information based on clinical experience with patients?* - *Did you reach out to health care providers in your provider network (e.g. a community of practice)?* |
|  | |
| **SECTION 2: EXPERIENCES AND PRACTICE CONTEXT OF HEALTH CARE PROVIDERS** | |
|  | |
| **“We are interested in learning about the practice context and your experiences as a health care provider who is involved in the management and treatment of patients with OUD…”** | |
| ***QUESTIONS*** | ***PROBES*** |
| How would you describe your role in the treatment and management of OUD? | - *Has your role changed over time? In what ways has it stayed the same? In what ways has it changed?* - *Has your role as a health care provider changed in response to the opioid crisis?* - *Does your role change depending on the patient you are treating?* |
| Could you take me through a typical day as a health care provider? | - *How long have you provided OUD care?* - *On average, how many patients do you interact with each day who have OUD?* - *What do you find most challenging about your job?* - *What do you find most rewarding about your job?* - *What types of things do you feel prepared for? What do you feel unprepared for?* |
| How would you describe your practice setting – that is, the place or places that you provide care to people with OUD? | - *Where is your office or practice located? Note* - *Do you feel that your work environment is supportive of evidence-based care for people with OUD?* - *If yes, what does this mean in your day-to-day practice?* - *If no, how do you manage this in your day-to-day practice? What changes would you make to improve the quality of care?* - *Do you feel supported by your colleagues? Why/why not? How does this impact the quality of care you provide for your patients?* |
| What do you feel is the overall goal of treatment for OUD? | - *How do you define this goal?* - *Who do you feel should define this goal?* - *How is the success of treatment defined?* |
| How would you describe the philosophy of care that you provide? | - *What guiding principles of care are followed (e.g., harm reduction, patient-centred care, integrated care)?* - *How do you integrate these principles into care?* - *Has your philosophy changed over time? How so?* |
| How would you describe the relationship you have with your patients with OUD? | - *Does this relationship differ depending on the patient?* - *Is it important for you to have a relationship with your patients? Why/why not?* |
| In general, how would you describe the impact that OUD treatment has had for your patients? | - *Impact on social and health outcomes (e.g., income, overall health, housing, emotional wellbeing, relationships with others etc...)?* - *Impact on engagement with health and social services?* - *Other outcomes/impacts you have noticed*? |
| Did you feel supported by your work when you attended the ECHO sessions? | - *If yes, how so? How does this impact your work?* - *If no, how does not feeling supported impact your work?* *Did you experience any pressures to not attend?* |
| Have you experienced any challenges from **your colleagues** in the context of OUD treatment and care? | - *Would you feel comfortable asking your colleagues for guidance on treating or managing a patient with OUD?* - *How does this impact the care you provide for your patients?* |
|  | |
| **SECTION 3: GAPS IN TREATMENT** | |
|  | |
| **“We are interested in learning about some of the challenges and gaps in treatment that health care providers encounter when managing and treating patients with OUD…”** | |
| ***QUESTIONS*** | ***PROBES*** |
| What are some of the main challenges you encounter in the clinical care setting when treating patients with OUD? | - *What makes it challenging?* - *Are these challenges unique to patients with OUD or do you encounter these problems with other patients as well?* - *Are there any policies in place that make it difficult to provide care?* - *How does stigma impact the relationship you have with your patients?* - Have you experienced any challenges from **the community** in the context of OUD? *If so, how did these challenges impact your practice?* - *Is there a perceived lack of community support for health care providers doing this work? If so, how does lack of community support impact health care providers and their patients?* - *Have you experienced any challenges from* ***institutional or governmental bodies****? If so, how did these challenges impact your practice?* - *Have you experienced any pressures or challenges from patient advocacy groups (e.g. safe supply prescribing, advocacy from the chronic pain community around opioid prescribing)? How did this impact your practice?* - *Is there pressure on health care providers from patient advocacy groups (e.g. safe supply prescribing, advocacy from the chronic pain community around opioid prescribing)? If so, how does this impact health care providers and their patients?* |
| What do you think are some of the factors that may influence or impact the treatment trajectory of a patient with OUD? | - *How do personal relationships impact the treatment trajectory?* - *How does a family history of substance use impact the treatment trajectory?* - *How does pain impact the treatment trajectory?* - *How do you think that these factors interact with each other?* |
| In your opinion, where does the responsibility lie in the management of OUD? | - *Who is responsible for managing OUD?* - *Is it an individual or collective responsibility? Why?* |
| In your opinion, how well do you think OUD is managed in your practice? | - *Do you think that the average health care provider is well equipped to manage and treat patients with OUD? Why/why not?* - *What gaps are there? How could these gaps be more adequately addressed?* - *What supports would be useful to help you provide care for people with OUD?* |
| Are there any barriers for patients to access OUD treatment and care in your practice setting? | - *If so, how could this be addressed?* - *Do you think that your practice setting is accessible to people with OUD? Why/why not?* |
| What are your experiences maintaining continuity of health care for your patients with OUD? | - *Is this different across pharmacies, hospitals, and clinics?* - *How do you ensure that you are meeting clients’ physical, emotional, spiritual needs?* |
| What is the size of the community you serve? How does the community size impact the care you provide for your patients? | - *Does it help or hinder care? Why?* - *What is it about these communities that makes it easy or hard?* - *Is it different from rural to non-rural?* - *Is there a stigma in this community towards people who use substances?* - *Are there training opportunities available for health care providers in the community?* |
| Where is your practice located? How does the geographic location of your practice impact the care you provide for your patients? | - *Does it help or hinder care? How so?* - *Is there anything that could be done to improve the quality of care in your geographic location?* - *Are there differences in access to resources/supports?* - *How does this impact the health of your patients in the community?* |
| In your opinion, what impact has the overdose crisis had on the way you treat and manage patients with OUD? | - *What role do health care providers play*   *in addressing the overdose crisis?*   - *In what ways do prescribing practices impact the overdose crisis?* - *How does OUD Project ECHO impact a health care providers ability to make a difference in the crisis?* - *How has the overdose crisis impacted your practice?* |
|  | |
| **SECTION 4: PERCEPTIONS OF OUD PROJECT ECHO** | |
|  | |
| **“We are interested in learning about your perceptions of OUD Project ECHO as a health care provider…”** | |
| ***QUESTIONS*** | ***PROBES*** |
| How did you first hear about Project ECHO? | - *Who told you about it?* - *What did they tell you about it?* - *What did you think about this information?* |
| What were your expectations about Project ECHO? | - *Did you have any reservations about participating? Why/why not?* |
| What was your motivation to enrol in Project ECHO? | - *Why did you want to participate?* - *What were the motivating factors?* - *Were you motivated by professional growth?* - *Did you have support from your employer?* - *Did you employer recommend that you attend the ECHO sessions?* - *Are there any other reasons you wanted to participate?* |
| What were your short-term goals while attending the Project ECHO sessions? | - *Anything you were hoping to learn?* - *Have you achieved those goals? Why/why not?* |
| What were your long-term goals while attending the Project ECHO sessions? | - *Anything you were hoping to learn?* - *Have you achieved those goals? Why/why not?* |
| What did you think about the content of the ECHO sessions? | - *In how many sessions did you participate?* - *How long ago was your last ECHO session?* - *Were there some sessions that you were more keen to attend? Which ones? Why?* - *Did you find the case studies and PowerPoint presentations useful? Why/why not?* - *Do you think the information in the ECHO sessions was evidence-based? Why/why not?* - *Did you find the content of the ECHO sessions relevant for your practice?* - *What topics would you like to see covered in the future?* |
| How did you feel about the format of the Projects ECHO sessions? | - *Did these formats meet your learning needs and preferences? Why or why not?* - *Is there a different format you would prefer?* - *Was the approach aligned with how you learn / retain information? Why or why not?* - *What did you think about the length of the sessions?* |
| How did you feel about the web interface platform, Zoom, used for Project Echo? | - *Do you think web interface is an effective medium to communicate information to health care providers? Why/why not?* - *What was your comfort level with using this platform? Did the platform create any barriers to attend the ECHO sessions?* - *Do you have any ideas of other platforms that would be more useful to run the ECHO sessions?* |
| In addition to the OUD ECHO, the BCCSU has launched a podcast for clinicians to learn about OUD treatment, have you listened to this podcast? | - *If so, what did you think of the podcasts?* - *Did they enhance your learning from the ECHO? Did they help improve your practice in any way?* - *If you haven’t listened to the podcast, why not?* |
|  | |
| **SECTION 5: IMPACT OF OUD PROJECT ECHO** | |
|  | |
| **“We are interested in learning about the impact of OUD Project ECHO…”** | |
| ***QUESTIONS*** | ***PROBES*** |
| Have the ECHO sessions impacted your practice in any way? | - *If* ***YES****, how have the ECHO sessions impacted your practice? Is there anything that you’ve implemented as a result of the ECHO sessions? What were some of the facilitators in your practice that allowed you to implement these changes? (e.g., supportive work environment, positive patient-physician relationships, etc.)* - *If* ***NO****, why have the ECHO sessions not led to any changes in your practice? What are some of the challenges to implementing these changes in your practice?* - *Have any of your prescribing practices changed as a result of Project ECHO?* |
| What skills or knowledge have you acquired as a result of attending the ECHO sessions? | - *How do you intend on applying the knowledge and skills you learned in the ECHO sessions in your practice?* - *Would you feel comfortable sharing this knowledge and information to other people in your clinical network of OAT?* |
| Which ECHO sessions were the most useful? | - *Why?* - *Which ECHO sessions were most engaging? In what ways?* |
| Which ECHO sessions were the least useful? | - *Why?* - *What could be improved to make them more effective?* |
| Have the ECHO sessions provided you with contextually relevant information for managing and treating patients with OUD? | - *If yes, how so?* - *If no, in what ways do you think the ECHO sessions aren’t contextually relevant for treating patients with OUD?* - *Do you feel that the recommendations made by ECHO are feasible in your setting? Why or why not?* - *How does this impact how you feel about the ECHO program?* - *Has your ability to access information related to OUD care changed since you began participating in the ECHO? How has it changed?* |
| Can you describe a scenario where something you learned in an ECHO session helped you in your practice? | - *What made this scenario challenging?* - *How would you have acted previously?* - *Are there any other scenarios where something you learned in an ECHO session helped you in your practice?* |
| Has your clinical network of OAT providers expanded or been strengthed at all since participating in the ECHO? | - *Do you have access to resources or connections to other OAT providers that you did not previously have?* - *If no, would you like to strengthen your network? How do you think that could be done?* - *If yes, in what ways have your connections expanded or been strengthened? What impact has this had on you and/or your practice?* |
| Have the ECHO sessions led to any changes in terms of social and physical supports in the community (e.g. knowledge of local resources or clinical tools)? | - *If yes, how have the ECHO sessions improved your social and physical supports? What impact has this had on the quality of care you provide for your patients?* - *If no, why is this the case? What impact does this have on the quality of care you provide for your patients?* - *Why is it important to have these supports in place for patients with OUD?* |
| How have the ECHO sessions impacted your understanding of the socio-structural factors that shape outcomes related to OUD and the overdose crisis? | - *How do you think about socio-structural factors as part of your assessment for OUD? How does this influence the care you provide?* - *Has participation in Project ECHO shaped the way you understand OUD conceptually?* |
| Have the ECHO sessions led to any changes in cost-savings? | - *Have the ECHO sessions led to sharing of clinical education, resources, and tools for opioid use disorder among your clinical network?* - *Have the ECHO sessions replaced or been an alternative to costly educational courses? Approximately how much do you think you are saving in comparison?* - *Do you think the ECHO sessions have led to improvements in OUD care in your practice and therefore, better outcomes in the community (e.g. less visits to the emergency, improved health of citizens in the community) resulting in cost savings?* - *Has the cost-savings from the ECHO sessions been re-allocated or re-invested in other areas?* |
| Has Project ECHO reduced duplication of similar OUD projects in the community? | - *If yes, how so?* - *If no, why do you think this is the case? What do you think needs to be changed to reduce the need for duplication of similar OUD projects?* - *Has Project ECHO developed and strengthened linkages with other organizations and partners reducing the need for other OUD projects in the community?* |
| Compared to June 2019 when the ECHO sessions began, how confident are you in your ability to manage and care for OUD patients? | - *What has changed over this period of time?* - *Have you gained more experience in OUD care and treatment?* - *Have you noticed treatment for patients with OUD changing provincially?* - *Did participation in the ECHO Project contribute to changes in your level of confidence?* |
| How well do you think that the ECHO sessions map on to different practice settings (e.g., pharmacy, hospital, clinics etc.)? | - *Do you think the echo sessions are relevant across different health care settings? Why/why not?* - *Do you think the echo sessions are relevant across different health care professions (e.g., nurse practitioner, pharmacist, registered nurse, etc.)? Why/why not?* |
| Is there anything you would change to improve the ECHO sessions? | - *If yes, what would you like to change? Is there a better way to develop a network for health care providers?* - *If no, is there anything that could be improved upon in the future?* - *What can Project ECHO do to better engage with community providers during the ECHO sessions?* |
| Do you think you’ll continue to use the information and lessons you learned in the ECHO sessions? | - *Why/why not?* - *Do you think the material from Project ECHO will have a lasting impact on your practice?* - *Do you think the relationships or connections that you developed as a result of Project ECHO will have a lasting impact on your practice?* - *Would you consider participating in a different future substance use disorder ECHO? (Why/why not)* - *Would you be interesting in attending some of the OUD sessions again in the next cycle? (Why/why not)* |
| Would you recommend OUD ECHO sessions to other health care providers? | - *In what situations do you think OUD Project ECHO would be helpful for providers?* - *In what situations would you not recommend the OUD ECHO?* - *Why/why not?* |
| Has Covid-19 impacted the way you manage and treat patients with OUD in your practice? | - *Were there any changes to your practice as a result of Covid-19?* - *What challenges did you experience in your practice? Did these challenges change over time?* - *In your practice, has Covid-19 impacted patient engagement with OAT treatment? In what ways?* - *Did the new clinical guidance for prescribers and pharmacists regarding OAT (released March 2020), have an impact on your practice? How so?* - *Did you utilize telehealth to treat patients with OUD? Why/why not? If yes, was this helpful? Why/why not?* |
| Did you attend any of the Covid-19 related ECHO sessions? | - *What were your thoughts on the ECHO session?* - *Did you find the ECHO session useful? Why/why not?* - *Did you apply any of the knowledge and skills you learned in the ECHO session to your practice?* - *Have any of your prescribing practices changed as a result of attending the Covid-19 ECHO session?* - *Would you feel comfortable sharing this knowledge and information to other people in your clinical network of OAT?* |

**Alcohol Use Disorder (AUD) Project ECHO Evaluation**

**Participant Interview Guide 2021**

**QUALITATIVE INTERVIEW COVER SHEET**

**FOR PARTICIPANTS REGARDING THEIR EXPERIENCE WITH**

**ALCOHOL USE DISORDER (AUD) PROJECT ECHO**

**Interviewer:**

**Date and Time:**

**File Name:**

**DEMOGRAPHICS – DO NOT TURN ON AUDIO RECORDER**

**1. What is your gender? Do you identify as:**

Man Woman Intersex Transgender Non-Binary

Two-spirit Prefer not to say Prefer to self-describe: _______________

**2. How old are you?**

**3. With which race or ethnicity do you identify?**

Indigenous / Aboriginal White (Caucasian) South Asian

East or Southeast Asian Black / African Canadian Other: _________________

**4. What is the highest level of education you have completed?**

Trade / technical training Bachelor’s Degree Professional Degree

Master’s Degree Doctorate Degree Other: ___________________

**5. What is your occupation?**

Physician Pharmacist Social Worker Registered Nurse

Nurse Practitioner Other: ___________________

**5a. (If a physician) what is your training background or specialty?**

Family medicine Internal medicine Psychiatry

Emergency medicine Preventative Medicine Other: _____________

**5b. If Family medicine, are you in a community-based longitudinal family practice?**  Yes No Unsure

**6. In which health care setting are you currently working?**

Emergency care Acute care Community care Other: __________

**7. Approximately how many patients with AUD do you have on your caseload?**

**8. How many years of experience do you have managing/treating patients with alcohol use disorder?**

< 1 year 1-2 years 3-4 years 5-10 years

10-20 years 20-30 years 30+ years

**9. In which health region are you currently working?**

Vancouver Coastal Health (VCH) Fraser Health (FHA)

Interior Health (IH) Vancouver Island Health Authority (VIHA)

Northern Health (NH) First Nations Health Authority (FNHA)

Yukon Territory Health Region Provincial Health Services Authority (PHSA)

Providence Health Care (PHC)

**10. How would you describe the geographic location of your place of work?**

Urban Rural Other: _____________________

**11. Before participating in the AUD Project ECHO, which of the following sources of evidence did you use to inform your AUD practice?**

UpToDate database Primary research liiterature

Provincial AUD guidelines Your clinical experience with patients

A formal community of practice Your informal professional network

BCCSU POATSP online learning modules Scientific conferences

The Addiction Care and Treatment Online Course (ACTOC)

Other sources (please list): Low Risk Alcohol Use Guidelines

**PROJECT ECHO (EXTENSION FOR COMMUNITY HEALTHCARE OUTCOMES)**

**Alcohol Use Disorder (AUD) Project ECHO Evaluation**

**Participant Interview Guide 2021**

**DIRECTIONS**

**TURN ON AUDIO RECORDER**

| **SECTION 1** | |
| --- | --- |
| **PREVIOUS AUD TRAINING & EDUCATION** | |
|  | |
| **“We are interested in learning about your previous training and education in alcohol use disorder prior to taking part in AUD Project ECHO…”** | |
| ***QUESTIONS*** |  |
| What degree or non-degree training or education have you received related to AUD?    e.g., Provincial Opioid Addiction Treatment Support Program, Addiction Care and Treatment Online Course, attended a seminar on AUD clinical guidance delivered by BCCSU or completed a fellowship in Addiction Medicine | • *As part of your entry-level health professional training?*  • *As part of your continuing professional development?*  o *Who offered it?*  o *What were your impressions of the training?* |
| How effectively did the training prepare you to provide care to people with AUD? |  |
| **SECTION 2** | |
| **EXPERIENCES & PRACTICE CONTEXT OF AUD HEALTH CARE PROVIDERS** | |
|  | |
| **“We are interested in learning about the AUD practice context and your experiences as a health care provider involved in AUD management and treatment…”** | |
| ***QUESTIONS*** | ***PROBES*** |
| How would you describe your experience with the treatment and management of AUD? | • *Would you consider AUD to be a NEW practice area you are taking on?*  • *How has your role in the treatment and management of AUD changed over time? In what ways has it stayed the same?*  • *What do you find most rewarding about AUD management?* |
| Did you feel supported by your workplace to attend the ECHO sessions? | • *Was your involvement in the ECHO sessions supported or recommended by your employer or organization? How does this impact your work?*  • *Did you experience any pressure to not attend?* |
| Where do you go for support related to AUD treatment and care? | • *How comfortable do you feel asking your colleagues for guidance on treating or managing a patient with AUD? How do you decide who to approach?*  • *Do you engage in team-based care (where physicians, specialists, nurses and other providers work together to provide AUD care)? If* ***YES****, what are the benefits of this approach? What are the drawbacks of this approach? If* ***NO****, what are the barriers to team-based care for you?*  • *What supports are you lacking?*  • *What learning or resource needs do you still have?* |
| How does the size of your community impact the care you provide for your patients? | • *Does it help or hinder care? Why?*  • *What is it about this/these community/ies that make(s) it easy or hard?*  • *What can be done to improve the quality of AUD care in your community or geographic region?*  • *What role does stigma play in your community with respect to people with AUD?* |
| **SECTION 3** | |
| **BARRIERS & FACILITATORS OF AUD TREATMENT** | |
|  | |
| **“We are interested in learning about some of the challenges or gaps in treatment, and the factors that enable best practices in AUD care…”** | |
| ***QUESTIONS*** | ***PROBES*** |
| What are some of the challenges you encounter when treating patients with AUD? | • *What makes it challenging?*  • *Challenges at the level of the individual patient?*  • *Challenges managing patients from specific populations (e.g. youth, seniors, South Asian population, Indigenous patients, etc.)?*  • *Challenges co-managing AUD and other substance use/health disorders?*  • *Challenges related to your colleagues or other health care providers?*  • *Organizational issues?*  • *Policies or regulations?*  • *Public opinion or perceptions?*  • *Are these challenges unique to patients with AUD or do you encounter these problems with other patients too?*  • *How have these challenges impacted your practice?* |
| Has Covid-19 impacted the way you manage and treat patients with AUD in your practice? | • *What challenges have you experienced during COVID-19? Did these challenges change over time?*  • *Has Covid-19 impacted patient engagement for AUD treatment? In what ways?*  • *Did you use telehealth to treat patients with AUD? Why/why not? If* ***YES****, was this helpful? Why/why not?* |
| Are there any barriers to patients accessing AUD treatment or care in your practice setting? | • *If* ***YES****, how could this be addressed?*  • *Do you think that your practice setting is accessible to people with AUD? Why/why not?* |
| What facilitates AUD care in BC? | • *What supports exist for health care providers working with patients with AUD?*  • *What enablers exist within your organization or health authority? Have you accessed them?*  • *What assists AUD care at the provincial or policy level?* |
| What are your experiences in maintaining continuity of care for your patients with AUD? | • *How do you ensure that you are meeting patients’ physical, emotional, spiritual needs?*  • *How do you ensure that your patients with AUD are supported across different settings (e.g. switching clinics, incarceration)?*  • *What could be done to improve continuity of care across practice settings?* |
| **SECTION 4** | |
| **PERCEPTIONS OF AUD PROJECT ECHO** | |
|  | |
| **“We are interested in learning about your perceptions of AUD Project ECHO…”** | |
| ***QUESTIONS*** | ***PROBES*** |
| How did you first hear about the AUD Project ECHO? | • *Who told you about it?*  • *What did they tell you about it?*  • *What did you think about this information?* |
| What would be the most effective ways to reach more providers across BC and Yukon, and engage them in joining the ECHO community of practice? | • *Which email lists, social media platforms, organizations, special interest groups, associations, or networks should we try to reach out to?*  • *What kind of messaging would resonate with providers treating or managing AUD? What about for providers treating or managing OUD?*  • *How would the messaging be different for addiction medicine specialists, family physicians, nurse practitioners or other groups?* |
| What was your motivation to register for an AUD Project ECHO session? | • *Why did you want to participate? (What were the motivating factors?)*  • *Did you have any reservations about participating? Why/why not?* |
|  | • *Are you considering participating in future ECHO sessions? (Why/why not)* |
| What did you think about the content of the AUD ECHO sessions? | • *Which session(s) were you more keen to attend? Why?*  • *Did you find the content relevant for your practice?*  • *Which sessions weren’t useful for you? Why?*  • *What learning needs for AUD care have been met for you, and which still remain?*  • *What topics would you like to see covered in the future?* |
| How did you feel about the format of the ECHO sessions? | • *Does the current format, with a lecture-style presentation, case presentation and discussion meet your learning needs and preferences? Why/why not?*  • *Did you feel comfortable speaking up during the sessions? Why or why not?* |
| How would you improve the ECHO sessions? | • *What can Project ECHO do to engage community providers more effectively during the sessions?*  • *How would you suggest we promote the ECHO evaluation surveys so that more participants respond to make the findings more meaningful for informing and improving our work?* |
| How are you finding the ECHO website in terms of its ease of use and the resources it offers? | • *Is it easy to use? What is confusing or unclear?*  • *How could the website, or the registration process be improved?* |
| Project ECHO sends out monthly newsletters to consenting registrants. Do you read the newsletters? | • *Why or why not?*  • ***If YES,*** *What is most valuable about the newsletters?*  • *How often do you think these newsletters should be sent out (e.g., monthly, more or lesss frequently)?*  • *How could the newsletters be improved?* |
| Project ECHO also has a podcast for clinicians to learn about OUD, and soon, AUD treatment. What did you think of the podcast? | • *Did they enhance your learning from the ECHO?*  • *Did they help improve your practice in any way?*  • ***If you haven’t listened to the podcast****, what is the reason?*  • *What would make the podcasts more appealing or effective for you?*  • *What topics would you like covered on the podcast?*  • *What would be the best way to raise awareness of the podcast?* |
| To what extent **is ECHO duplicating** similar AUD care supports or addressing existing gaps in BC? | • *What do you think needs to change to reduce duplication?*  • *What gaps has it addressed?*  • *What gaps remain?* |
| **SECTION 5** | |
| **PROVINCIAL AUD GUIDELINES** | |
|  | |
| **“We are interested in learning about your experiences with the provincial AUD guidelines…”** | |
| ***QUESTIONS*** | ***PROBES*** |
| Have you reviewed the provincial AUD guidelines that were developed by the BCCSU? | • *If* ***YES****, have the guidelines been helpful in your practice setting? Have you implemented anything from the guidelines into your practice?*  • *If* ***NO****, why have you not accessed them? What would be helpful to support your access or use of the guidelines?* |
| **If they accessed the guidelines:** What challenges, if any, have you had in understanding the recommendations in the provincial guidelines? | • *Are there any specific recommendations that were unclear or difficult to interpret? [show the recommendations on the screen]*  • *Has your learning through ECHO helped you to interpret the guidelines? Could you elaborate? e.g., which section of the guidelines; what was the misinterpretation, and what was done through ECHO to help clarify it for you?* |
| What challenges, if any, have you had in implementing the recommendations from the provincial guidelines in your practice? | • *What would make it easier for you to implement the recommendations?* |
| What is missing from the provincial guidelines? | • *Are there any content areas, resources or tools that would be beneficial to add to support you in providing evidence-informed AUD care?* |
| How should the national AUD guidelines that are being developed right now be implemented? | • *What strategies would be effective for disseminating the guidelines?*  • *What strategies would be effective for helping health care providers understand and apply the guidelines’ recommendations?*  • *How likely would you be to join a* ***national*** *community of practice about AUD, OUD or other substance use-related topics? What are the reasons for this?* |
| **SECTION 6** | |
| **IMPACT OF AUD PROJECT ECHO** | |
|  | |
| **“We are interested in learning about the impact of AUD Project ECHO…”** | |
| ***QUESTIONS*** | ***PROBES*** |
| What **knowledge or skills** have you acquired as a result of attending the ECHO sessions? | • *What was your biggest ‘aha’ moment from the ECHO sessions?*  • *Has your ability to access information related to AUD care changed since you began participating in the ECHO? How has it changed?*  • *Has your attitude toward patients or toward AUD care changed in any way?* |
| To what extent are the recommendations made by ECHO **feasible** in your setting? | • *What makes them un/feasible?* |
| How do you **intend to apply** the knowledge and skills you learned in the ECHO sessions in your practice? | • *How do you plan to change your practice after attending ECHO sessions?* |
| Is there anything that you’ve **already implemented** as a result of the ECHO sessions? | • ***If YES,*** *What have you implemented? What were some of the facilitators in your practice that allowed you to implement these changes? (e.g., supportive work environment, positive patient-provider relationships, etc.)*  • *If* ***NO****, why have the ECHO sessions not led to any changes in your practice? What are some of the barriers to implementing these changes?* |
| Can you describe a **scenario**where something you learned in an ECHO session helped you in your practice? | • *What made this scenario challenging?*  • *How would you have acted previously?*  • *Are there any other scenarios where something you learned in an ECHO session helped you in your practice?* |
| To what extent have you been able to **sustain the changes** you’ve made as the result of ECHO? | • *What are the barriers to sustainability?*  • *What makes/would make sustainability possible?* |
| Has your clinical **network expanded** or been strengthed at all since participating in the ECHO? | • *Do you have access to resources or connections to other providers that you did not have before?*  • *If* ***YES****, in what ways have your connections expanded or been strengthened?*  • *If* ***NO****, would you like to strengthen your network? How do you think that ECHO could be improved to support clinical network expansion more effectively?*  • *What impact do you think relationships or connections developed through ECHO have or will have on your practice? How long do you think these impacts will last?* |
| Have the ECHO sessions led to any **changes in terms of sharing**clinical education, resources and tools, or social or other practice supports in the clinical community? | • *If* ***YES****, what kinds of changes have you seen? What impact has this had on the quality of care you provide?*  • *If* ***NO****, why is this the case? What impact does this have on the quality of care you provide for your patients?* |
| Has your **confidence** in your ability to manage and care for AUD patients changed as a result of joining ECHO? | • *What has changed over this period of time?* |
| What impact has Project ECHO had on addressing challenges in care for, or in meeting the care needs of special populations? | • *(e.g. youth, seniors, South Asian population, Indigenous patients, etc.)?* |
